# Dual manipulation of individuals by non-invasive intelligent technologies and its risk governance: from the perspective of the Destiny Machine metaphor

**DOI:** 10.3389/fpsyg.2026.1949628

**Published:** 2026-10-28

**Authors:** Qinqin Wang

**Affiliations:** 1 School of Humanities, Southeast University, Nanjing, China; 2 AI Ethics Lab, Southeast University, Nanjing, China

**Keywords:** Destiny Machine, ethical risks, human-centered approach, psychological mechanisms, technological manipulation

## Abstract

**Introduction:**

The “Destiny Machine” metaphor reveals an ethical concern: big data and artificial intelligence, operating as a data-power social machine, manipulate individuals with pre-determinacy as the core logic. At cognitive-information and behavioral decision-option levels, this manipulation appears as cognitive manipulation by recommendation algorithms and decision-making manipulation by automated decision systems. To uncover its psychological mechanisms and ethical risks, this paper builds a framework integrating ethics and psychology.

**Methods:**

The Destiny Machine is transformed into an analytical tool with three dimensions: pre-determinacy, irresistibility, and concealment. Drawing on dual-process theory, automation bias, moral disengagement, and psychological reactance, it analyzes how non-invasive intelligent technologies psychologically manipulate individuals and shape their ethical risks, adopting the human-centered approach as the guiding principle for governance.

**Results:**

Cognitive manipulation by recommender systems, typified by TikTok, leaves users unaware that their cognitive preferences are being systematically reshaped, keeping them imperceptibly immersed. Algorithms achieve this by activating Type 1 processing while bypassing Type 2 scrutiny, confining information to relevance channels, and exploiting automation bias and complacency to lower cognitive vigilance and suppress psychological reactance. Indulging in information cocoons widens users’ cognitive distance from the lifeworld, dissolving their moral attributes as ethical beings. Decision-making manipulation by automated decision systems is most evident in autonomous vehicles’ trolley-problem dilemmas, where passengers’ lives are reduced to quantifiable weights. The system pre-sets decision rules and allows remote intervention at the design end; opaque decision-making subjects and logic, millisecond-level deadlines, and impossibility of exit jointly pre-lock passengers’ fate before the accident occurs. It likewise exploits cognitive processing traits and automation bias, while dispersed agency makes responsibility attribution—and thus psychological reactance—difficult. Pre-coding an individual’s fate at the behavioral decision-option level may deconstruct their meaning world.

**Discussion:**

Under the Destiny Machine metaphor, such manipulation erodes not only individuals’ autonomy but also their responsibility as moral subjects. Guided by the human-centered approach, it proposes governance schemes with clear actors, sound measures, and reliable benchmarks: a cognitive immune system of transparency and empowerment for cognitive manipulation, and a human-machine collaborative scheme of accountability and remedy for decision-making manipulation, rebuilding passengers’ trust to help them escape.

## 1 Introduction

Algorithmic manipulation has become a major issue in digital ethics. Scholars debate its concept, mechanisms, and ethical risks, yet no consensus on its definition, operation, or harms. Three representative approaches have emerged. The intention-oriented approach anchors manipulation in the explicit malicious intent of the agents behind algorithms (Ienca, 2023). The structural-power approach treats it as a disciplinary technique of power within algorithmic governance (Wang, 2022). The attitude-oriented approach holds that manipulation need not stem from malicious intent but may arise from designers’ indifference to users’ rational autonomy (Klenk, 2023). Synthesizing these perspectives, this paper defines algorithmic manipulation as a power technique disciplining and regulating users’ cognition and behavior, with underlying agents harboring explicit or implicit intent, strategically manifesting as designers’ indifference to users’ rational autonomy.

The three approaches above account for algorithmic manipulation in different ways, but understanding how such manipulation erodes individual subjectivity requires moral psychology’s theory of mind perception. Drawing on large-scale surveys, this theory shows that people perceive others’ minds along two dimensions: agency and experience. Agency is the capacity to plan, act, and intend, whereas experience is the capacity for feeling, emotion, and suffering. These dimensions correspond to two moral types: agents, characterized by agency, are held morally responsible, while patients, characterized by experience, are entitled to moral rights and protection (Gray et al., 2012).

Mind perception provides the moral-psychological basis for understanding algorithmic manipulation’s ethical substance: when algorithmic systems reduce users to data points rather than experiencing subjects, they deny their capacity, as moral patients, to feel pain and suffer harm. Gray et al. (2012) link deficits in mind perception directly to moral judgment: without perceived agency, one struggles to understand moral agents; without perceived experience, one struggles to understand moral patients. Thus, when algorithmic systems use information asymmetry, black-box decision-making, or neural surveillance to covertly and continually discipline and regulate users, they structurally erode agency and experience—the two capacities that make users full moral subjects. Across these approaches, the ethical harm of algorithmic manipulation lies in weakening, even denying, users’ status as both moral agents and moral patients.

On mechanisms, some scholars treat information asymmetry as a precondition for algorithmic manipulation, which, through big-data hypernudging, continually refines its influence pathways (Yeung, 2017). Social media recommendation algorithms continually test content presentation to maximize engagement, potentially trapping users in information cocoons (Sass, 2024). Others stress that black-box opacity is a key source of manipulative potential: relying on massive datasets and complex models, algorithmic systems hide their decision-making, making it difficult for individuals to assess or resist manipulation (Sahakyan et al., 2025). Drawing on neurotechnology advances, some scholars argue that algorithmic intrusion into consciousness constitutes a deeper mechanism of manipulation (Drew, 2025).

Three representative views on ethical risks exist. First, platform-based algorithmic manipulation erodes users’ autonomy (Graff, 2026). Second, AI-driven neurotechnology extends algorithmic manipulation risks from external environments into the brain, creating ethical challenges in human-machine symbiosis—system dependence and even addiction, self-perception biases, and identity crises (Wu and Chen, 2025). Third, algorithmic manipulation’s corrosion of independent judgment and public rationality will be exponentially amplified by generative AI development (Susser et al., 2019).

Against this backdrop, data ethicist Hasselbalch (2021) proposes the Destiny Machine metaphor to reveal the data-power social machine that leads, guides, and defines human life in the big data society, and the ethical challenges it creates. This metaphor offers insight into how non-invasive intelligent technologies manipulate individuals. Drawing on this metaphor and Fabbri’s (2025) system-agent-patient (S-A-P) framework, this paper examines manipulation of two commercially deployed algorithmic systems: recommender systems (RS, where A = P and S ≠ A) and automated decision-making systems (ADMS, where S = A and A ≠ P). First, following the metaphor’s pre-determinative logic, it builds a psychologically grounded three-dimensional framework for analyzing non-invasive technological manipulation—pre-determinacy, irresistibility, and concealment—and clarifies the boundaries of such manipulation. Second, it applies this framework to TikTok’s recommender system and self-driving cars’ automated decision-making system, identifying their manipulation types and underlying psychological theories. Third, it assesses the ethical risks these two forms of algorithmic manipulation may pose from the perspective of the ethical mode of human existence. Finally, guided by the human-centered approach—which holds that humans in the era of digital intelligence should actively assume ethical responsibility—it proposes targeted, feasible governance strategies for each form of manipulation and its ethical risks.

## 2 Analytical tools and applicable boundaries for technological manipulation under the Destiny Machine metaphor

In a big data and AI society, Hasselbalch’s Destiny Machine metaphor is essentially a warning that intelligent technology as a distinctive sociotechnical power may manipulate individual lives. Here, intelligent technology’s manipulation of individuals rests on a new contractual form of intensified surveillance, relies on data extraction and analysis, and features continuous experimentation and personalization (Zuboff, 2015). A systematic analysis of technological manipulation requires both turning the descriptive Destiny Machine metaphor into a mature theoretical-analytical tool and clarifying the applicable boundaries of intelligent technological manipulation—specifying, in particular, what uses of intelligent technology do not constitute risks of manipulation.

### 2.1 Technological manipulation in the Destiny Machine metaphor

In the surveillance society of the digital-intelligence era, Big Data Socio-Technical Infrastructures (BDSTIs) and Artificial Intelligence Socio-Technical Infrastructures (AISTIs) constitute a distinctive form of technological power in the digital surveillance society. BDSTIs operate through the digitization of everyday life; AISTIs are an upgraded iteration, entering daily life through data management and shaping human past and present by concretizing visions of the future (Hasselbalch, 2021, p. 11). Hasselbalch introduces the “Destiny Machine” metaphor to describe technological systems and processes designed to predict human behavior based on the accumulation of personal data and then act on these predictions—machines that produce machine-readable people and shell out destinies (Hasselbalch, 2021, p. 28). This reveals the top-down ways in which intelligent technologies frame human individuals and their futures.

Under the Destiny Machine metaphor, intelligent technologies’ manipulation of individuals (hereafter technological manipulation) must meet these conditions. First, its medium must be internet-based intelligent connectivity. Second, it must rest on accumulation and analysis of individuals’ behavioral data and apply predictive analytical results to the original target, while automatically adjusting data model parameters to achieve increasingly precise predictive inducement. Third, it must be grounded in individuals’ past and present lives and directed toward their future lives. Technological manipulation in the Destiny Machine sense is represented at the cognitive-informational level by recommender systems and at the behavioral-option level by automated decision-making systems. Though the two types of algorithmic manipulation differ sharply in physical implementation, they share the pre-determinacy logic of the Destiny Machine metaphor.

### 2.2 Theoretical analytical tools for technological manipulation

Clearly, the Destiny Machine is a critical metaphor revealing how BDSTIs and AISTIs reconfigure social power in the digital-intelligence era; it cannot directly serve as an analytical tool for technological manipulation. To overcome this, this paper follows the pre-determinative logic of the Destiny Machine metaphor and proposes three analytical dimensions grounded in psychological theory—pre-determinacy, irresistibility, and concealment—to transform the Destiny Machine from a metaphorical descriptive framework into a mature theoretical-analytical tool.

Pre-determinacy refers to the extent to which intelligent technologies, through accumulation and analysis of users’ behavioral data, pre-set or constrict future action trajectories before the user acts. In the philosophical tradition of “ism of ‘doing’” (Ma and Jing, 2003), its defining characteristic is linear extrapolation from past to future rather than critical reflection. It directly addresses the extent to which technological systems pre-determine users’ destiny. Irresistibility refers to the difficulty and cost incurred by users when attempting to deviate from or resist pre-set action trajectories. It directly addresses whether individuals possess the capacity to resist within such systems, and what hidden costs resistance entails. Concealment concerns whether users are aware that intelligent technologies are pre-determining their future life trajectories. The non-transparency of this process addresses whether users are informed about the manipulation they face—when unaware, the precondition for consent or resistance disappears.

From a psychological perspective, these three dimensions are linked, to varying degrees, to automation bias, dual-process theory, moral disengagement, and psychological reactance. Automation bias (Mosier et al., 1996) is a systematic tendency driven by cognitive miserliness, trust in authority, and diffusion of responsibility. It overlaps heavily with “automation complacency,” is moderated by task load, system reliability, and individual differences, and is difficult to eliminate through simple training or prompts (Parasuraman et al., 1993; Parasuraman and Manzey, 2010).

Dual-process theory (Kahneman, 2011) distinguishes two types of cognition: Type 1 (autonomous, fast, requiring no working memory) and Type 2 (controlled, slow, requiring working memory) (Evans and Stanovich, 2013). They interact through a default-interventionist mechanism, moderated by cognitive capacity, motivation, time pressure, and situational features.

Moral disengagement, proposed by Bandura (1990) to explain how individuals selectively disengage moral self-sanctions from inhumane conduct, is the process of deactivating self-imposed moral constraints through mechanisms such as cognitive restructuring, displacement of responsibility, ignoring consequences, and devaluing victims, making unethical behavior psychologically acceptable to themselves (Bandura, 1999, 2016).

Psychological reactance theory (Brehm, 1966; Brehm and Brehm, 1981) holds that when free behaviors are threatened or eliminated, people enter a motivational state to restore freedom. Reactance intensity depends on factors such as the importance of the freedom, threat severity, and restoration difficulty (Brehm and Brehm, 1981; Rosenberg and Siegel, 2018). Reactance can manifest as increased attraction to the threatened freedom, hostility and source derogation, or a boomerang effect. When restoration fails, individuals may shift toward learned helplessness (Rosenberg and Siegel, 2018).

Using the differing explanatory power of the psychological theories for the three analytical dimensions of technological manipulation, this paper identifies five categories—core mechanism, reinforcing mechanism, boundary condition, weak association, and suppression—summarized in Table 1. The following section uses it to systematically explain the linking mechanisms, triggering conditions, and applicable boundaries between each dimension and the four theories.

**Table 1:** A psychological theory-based analytical tool for technological manipulation.

Analytical dimension	Automation bias	Dual-process theory	Moral disengagement	Psychological reactance
Pre-determinacy	▲	▲	◆	■
Irresistibility	●	◆	●	▲
Concealment	●	●	▲	✕

▲ = Core mechanism (primary driving role); ● = Reinforcing mechanism (synergistic amplification effect); ◆ = Weak association (requires joint operation with other dimensions); ■ = Boundary condition (determines when the dimension becomes ineffective or triggers resistance); ✕ = Suppressed (cannot be activated due to the characteristics of the dimension). All labels are left-aligned within cells.

#### 2.2.1 The pre-determinacy dimension

In technological manipulation, pre-determinacy has automation bias and dual-process theory as core mechanisms, psychological reactance as its boundary condition, and a weak association with moral disengagement.

Specifically, When users trust automated aids and perceive them as possessing superior analytical capabilities and authority, they may overestimate the aids’ performance and use automated outputs as a “heuristic replacement” for vigilant information seeking and processing, thereby giving rise to automation bias (Parasuraman and Manzey, 2010). Cognitively, system-preset information flows, default options, and decision paths are more likely to directly activate Type 1 autonomous processing, while Type 2, following the law of least effort, usually adopts Type 1’s choices without review. In particular, when working memory load is high or motivation/rational thinking dispositions are insufficient, the likelihood that Type 2 processing intervenes in Type 1 default responses is further reduced (Evans and Stanovich, 2013).

Dual-process theory provides a solid psychological basis for analyzing how intelligent technologies shape user decisions through preset options. If system presets are perceived as forcibly depriving users of choice, psychological reactance may trigger an intrinsic motivation to restore freedom; when restoration is moderately likely, such presets lose their effect (Brehm and Brehm, 1981; Rosenberg and Siegel, 2018). This theory offers a motivational framework for how preset options in intelligent technologies provoke resistance or are suppressed. Moral disengagement, though weakly associated with pre-determinacy—which alone does not sever causal responsibility but works with concealment—may be indirectly reinforced when users cannot attribute responsibility.

#### 2.2.2 The irresistibility dimension

As an indispensable dimension of technological manipulation, irresistibility has psychological reactance as core mechanism, automation bias and moral disengagement as reinforcing mechanisms, and a weak association with dual-process theory. According to psychological reactance theory, when a technological system’s exit costs are extremely high and its ecosystem lock-in makes restoring freedom nearly impossible, users’ initial resistance is forced to turn into learned helplessness or passive compliance—i.e., acceptance of ecosystem lock-in. In this process, automation bias and moral disengagement play reinforcing roles. Specifically, on the one hand, users tend to shift monitoring and decision-making responsibility to the technological system and cease to intervene actively, which may lead to errors of omission and commission (Skitka et al., 1999); on the other hand, when users feel they have no choice within the technological system, they actively shirk responsibility, which objectively provides a moral excuse for technological manipulation. As for dual-process theory, the irresistibility of technological systems may indirectly reduce the probability of Type 2 intervention by depleting cognitive resources or suppressing the motivation to restore freedom.

#### 2.2.3 The concealment dimension

As another analytical dimension of technological manipulation, concealment has moral disengagement as core mechanism, automation bias and dual-process theory as reinforcing mechanisms, with psychological reactance suppressed. Specifically, on the one hand, systems based on BDSTIs and AISTIs sever the causal chain of responsibility through the algorithmic black box while projecting a veneer of “technological neutrality,” leaving users unable to attribute responsibility, so manipulation can occur in an ethical vacuum. On the other hand, when perceived as constant and reliable, they induce premature cognitive commitment and complacency (Parasuraman et al., 1993), cognitively lowering users’ rational guard. Moreover, the technological ideology of “what you see is all there is” further blinds users, leaving no cues to activate Type 2. By preventing users from perceiving threats to freedom, systems suppress psychological reactance (Brehm and Brehm, 1981), reducing resistance and securing acceptance of system outputs; the influence may thus remain unperceived as a threat to freedom, even if not wholly imperceptible.

As shown, the same psychological theory plays different roles across analytical dimensions, and its cross-application involves functional differences and conditional constraints. This cross-adaptation is not mechanical correspondence but functional integration based on each dimension’s triggering conditions and theoretical boundaries. It systematically presents the multiple connections between the analytical dimensions of technological manipulation and psychological theories and lays a theoretical foundation for later analysis of the specific mechanisms of cognitive and decision-making manipulation.

### 2.3 Clarifying the applicable boundaries of technological manipulation

Technological manipulation reveals a vision of technology dominating humanity. Suartama et al. (2026) classify current human-AI relationships in education into three paradigms: AI-directed, AI-supported, and AI-empowered, differing not by the tool but by the learner’s cognitive role. Notably, in the AI-directed stage, students passively follow system instructions and are thus most likely to approach the boundary of technological manipulation. In the AI-empowered stage, by contrast, students are positioned as “Human in the Loop,” able to strategically guide AI in task execution and focus on higher-order cognitive activities such as critical evaluation, synthesis, and creative guidance. This strongly safeguards human agency and genuine autonomy, helping prevent technological manipulation. It follows that the technological manipulation discussed here belongs to the AI-directed paradigm.

It must be acknowledged that although the three dimensions above are appropriate for analyzing technological manipulation, any metaphor has explanatory limits. To avoid overgeneralizing, this paper specifies that a non-invasive intelligent technological system does not constitute Destiny Machine manipulation when it meets these conditions: (1) its operation is fully transparent and explicable, as with open-source algorithms and real-time queryable decision logic; (2) users have a complete, low-cost right to exit, e.g., disabling all personalized recommendations with one click without loss of functionality; and (3) users can deviate from preset paths without penalty, e.g., refusing personalized recommendations without experience degradation. Moreover, user resistance and the AI-supported and AI-empowered paradigms cannot be overlooked. By improving digital literacy, using countermeasures such as de-recommendation browser extensions, and pursuing legal empowerment through the right to object to solely automated decision-making (see Regulation (EU) 2016/679, 2016; Article 22), humans can to some extent resist and even neutralize technological manipulation.

## 3 Ethical risks and governance of cognitive manipulation

TikTok, a commercial social media platform, has over one billion users worldwide (Deng et al., 2024); its recommendation algorithm has become increasingly precise and responsive through real-time interaction and feedback with a massive user base. Under Fabbri’s (2025) SAP framework, it is a recommender system (RS). The algorithm first caters to users’ cognitive needs to lower vigilance, then gradually narrows information sources and scope, expanding control and dominating the information flow; users typically remain passive recipients. From the Destiny Machine perspective, this “cater first, control later” mode is precisely an excessive application of the AI-directed paradigm (Suartama et al., 2026) to commercial recommendation algorithms, i.e., induced cognitive manipulation like the “boiling frog” effect. As reliance on algorithmic output deepens, users may experience gradually widening cognitive social distance from the lifeworld and steadily declining willingness for autonomous moral reasoning and judgment, raising the ethical risk of dissolving their moral attributes as ethical beings. Governance requires a cognitive immune system with clearly assigned responsible actors and sound governance strategies.

### 3.1 Cognitive manipulation by recommender systems

Cognitively, the Destiny Machine metaphor’s technological manipulation is best exemplified by TikTok’s recommender system. It analyzes user-user and content-content correlations to derive a correlational recommendation algorithm (Jiang et al., 2025). Through data collection, profiling, and precise feeding (Xu et al., 2022), it tailors “information cocoons” (Sunstein, 2006) for each user. Specifically, the algorithm reinforces filter bubbles and echo chambers, narrowing cognition and inducing a constrained mindset, influencing users via subtle nudges, not commands (Fulmer, 2026). Through pre-determinacy, irresistibility, and concealment and their psychological mechanisms, this essentially constitutes cognitive manipulation by recommender algorithms.

First, pre-determinacy in recommendation algorithms shifts from predicting preferences to pre-determining trajectories. Using collaborative filtering and deep neural networks, they continuously collect and analyze browsing data—viewing time, likes, shares—to build preference profiles, preset information entry agendas, confine information to pre-calculated relevance channels, and generate order and visibility boundaries of the information flow before users search. Users appear to browse freely, yet their cognitive boundaries are algorithmically locked. Dual-process theory (Evans and Stanovich, 2013) distinguishes Type 1, operating automatically and quickly with minimal working memory, from Type 2, requiring attention and avoiding unnecessary exertion per the law of least effort. Recommendation algorithms primarily activate Type 1 while bypassing Type 2’s scrutiny—complex computation, deliberate reasoning, self-control—revealing how potential cognitive manipulation becomes actual.

Second, the irresistibility of recommendation algorithms manifests: leveraging automation bias and the complacency effect, the algorithm continuously feeds users content aligned with their preferences. While catering to cognitive needs, it subtly lowers cognitive vigilance and suppresses reflective scrutiny. Though cognitive dissonance arises, psychological reactance is suppressed, making users more likely to accept algorithm-curated paths and more willing to outsource the massive, complex task of information filtering to recommender systems. By ceding control over information filtering, users gradually lose the motivation and capacity to explore diverse information, while their cognitive dependence deepens. Eventually, cognitive structures rigidify, and users develop uncritical, blind acceptance of recommendation algorithms.

Finally, concealment in recommendation algorithms manifests as users’ imperceptible immersion and tendency to see content as personally interesting rather than what the algorithm deems they should be. Recommendation mechanisms are deeply embedded in daily information interaction, yet their logic remains obscured by the black box. Users often fail to notice that their cognitive preferences are systematically reshaped. This concealment turns manipulation from explicit coercion or persuasion into a seemingly neutral construction of information environment. While enjoying personalized recommendations, users are likely to overlook the algorithm’s value intentions and, without critical reflection, directly accept the algorithmically curated cognitive world.

In theory, users can partially resist through privacy modes, cross-platform verification, or disabling personalized recommendations. However, such resistance faces three structural costs: first, most users remain in technological unconsciousness (Zhou and Luo, 2025), unfamiliar with how recommendation algorithms work, thus struggling to form psychological motivation to resist; second, research confirms users typically prioritize short-term benefits of non-resistance over those of resistance, such as saving time and money (Bonini and Treré, 2024, p. 25); third, the rapid iteration and black-box nature of algorithms typically increase or even overload cognitive load, making resistance typically short-lived and fragile (Bonini and Treré, 2024, p. 44).

### 3.2 Cognitive social distance: risk of dissolution of moral attributes

Under the Destiny Machine metaphor, recommendation algorithms’ information cocoons stem from their “cater first, control later” logic, erecting an increasingly narrow and closed cognitive wall between users and the external information world. Continuously reinforced, this wall creates an information world of severe cognitive bias. Psychologically, the algorithm exploits cognitive miser tendencies, fueling mental inertia (Fulmer, 2026). Technically, this biased world is the algorithm’s encoding of users via data decoding. Ethically, confining individuals’ cognition of interpersonal and social relations within data cages via information cocoons, isolating them from the diverse lifeworld, algorithms affect their grasp of these relations, generating social distance in moral cognition. As Zygmunt Bauman argues in Chapter 7 of Modernity and the Holocaust, moral inhibitions are inseparable from proximity between people; as social distance increases, it becomes easier to commit immoral acts (Bauman, 1989, Chapter 7). Thus, moral concepts and ethical principles are rooted in social production and lived practice; as people gradually distance themselves from interpersonal interaction, their moral perception becomes increasingly dull, and the line between moral and immoral action increasingly blurred.

In this regard, insofar as users’ cognition is confined within data cages by algorithms, this further fuels automation bias and over-reliance on system output (Fulmer, 2026). It follows that when facing complex or even dilemmatic moral situations, users may lean more toward algorithms’ so-called optimal solutions, with little willingness for autonomous moral reasoning and judgment. Such solutions, however, often maximize efficiency, interests, or particular values while neglecting or even discarding moral emotions such as the duty of love, esthetic pleasure, and conscience. Moreover, as individuals gradually slacken in autonomous moral reasoning and judgment yet increasingly rely on algorithmic output, moral disengagement tends to occur cognitively.

In sum, under the cognitive manipulation of recommendation algorithms, users not only become more immersed in the biased cognitive world of information cocoons, but their moral disengagement is also reinforced by algorithmic systems, while their awareness and capacity for autonomous moral judgment and value choice gradually deteriorate. Thus, their immoral behavior may even be rationalized. This further confirms Bauman’s judgment: social distance makes immoral acts easier. Ethically, this risks dissolving the intrinsic nature of “what makes a human human” (Cheng, 2022)—namely, humans’ moral attributes as ethical beings.

### 3.3 Building a cognitive immune system combining transparency and empowerment

Under the Destiny Machine metaphor, cognitive manipulation by recommendation algorithms and its ethical risks erode cognitive capacity and disregard responsibility as ethical beings. The human-centered approach holds that humans, as ethical beings, are responsible to humanity and to life and existence broadly, advocating an empowering human infrastructure (Hasselbach, 2024, p. 5). This provides a proactive path for governing technological manipulation and its ethical risks. For recommendation algorithms, their adaptive closed-loop ecosystem uses a funnel-based model—recall layer, ranking layer, supplementary strategies, and algorithm layer (Wang, 2020, p. 13)—to efficiently filter relevant content. In practice, it is opaque and monopolizes information content. To effectively govern such cognitive manipulation and its ethical risks, we propose a cognitive immune system combining transparency and empowerment, with clear responsible actors and sound governance measures as requirements.

First, algorithm developers should provide plain-language explanations of recommendation mechanisms—data types used (Regulation (EU) 2016/679, 2016; Article 4(4)), private-data handling (Regulation (EU) 2016/679, 2016; Article 17), and non-profiling options (Regulation (EU) 2022/2065, 2022; Article 38)—and establish a compliance rate benchmark for algorithm transparency assessment reports. This helps users understand recommender system operation, discern recommendation intentions, and enhance algorithmic transparency. Importantly, transparency here means not full model disclosure but a layered, functional principle. It also requires platform operators to clearly inform users on the interface that algorithmic personalized recommendations influence the information types and scope they perceive.

To measure compliance rates of algorithm transparency assessment reports, we draw on the TOROS scale (Transparency Of RObots Scale) developed by Angelopoulos et al. (2025), adapting its three factors—Illegibility, Explainability, and Predictability—as items for recommendation algorithm transparency. TOROS is the first standardized, psychometrically validated scale of robot transparency, showing high cross-linguistic reliability and convergent validity across three cross-national samples (*N* = 1,223). Notably, TOROS originally measured perceived transparency of robot behavior; we adapt its items to recommendation algorithms, and this transfer’s applicability requires further examination in future research.

Second, platform operators should work with algorithm developers to instrumentally empower users via filtering tools—adjusting interest tag weights, blocking content sources, disabling personalized recommendations—granting autonomous choice and intervention over recommended content and support for deviating from preset paths. This empowerment substantively responds to users’ right to know, enabling dynamic information boundaries and reconstructing cognitive subjectivity, turning passive receivers into active managers. Crucially, it is not only an external institutional arrangement but a mechanism activating psychological defense by enhancing self-efficacy. Bandura (1997) defines self-efficacy as beliefs in one’s ability to organize and execute behaviors for desired goals, affecting persistence and coping. High self-efficacy individuals favor problem-focused coping over passive avoidance. Thus, when instrumental empowerment genuinely enhances users’ control over algorithmic systems, their self-efficacy substantially improves, further activating rational reflection and critical awareness, prompting them to actively identify algorithmic biases and take effective resistance.

Given information cocoons’ constraints on users’ information flows, the information source diversity index serves as an important benchmark for judging whether users have actually obtained instrumental empowerment. This index can be operationalized using the diversity dimension of Wang X. et al.’s (2025) multidimensional measures of information acquisition. That dimension uses entropy to calculate the evenness of information acquisition across sources, with higher entropy indicating greater source diversity. The scale was validated through a five-wave longitudinal survey (*N* = 698) and a two-wave cross-topic survey (*N* = 969), showing good test–retest reliability and convergent, discriminant, and concurrent validity.

Finally, an independent third-party regulatory body should be established for recommendation algorithm systems, comprising government, technical experts, ethics risk assessment experts, and user representatives. This body should rigorously assess and monitor platform operators’ compliance with legal provisions on privacy data protection, non-profiling recommendation options (Regulation (EU) 2022/2065, 2022; Article 38), and non-deceptive technologies (Regulation (EU) 2024/1689, 2024; Article 5(1)(a)). The four standards—fairness, diversity, controllability, and addictiveness (See Cyberspace Administration of China Ministry of Industry and Information Technology Ministry of Public Security State Administration for Market Regulation, 2024)—form the basic benchmark for commercial recommender system compliance. National education systems should also develop integrated digital literacy education across primary, secondary, and tertiary levels, focusing on information authenticity, algorithmic logic, and data ethics. Through systematic curricula and practical training, this education should substantively enhance citizens’ digital literacy cognitively and behaviorally, enabling independent judgment and rational criticism in using recommendation algorithms. Governments should then work with algorithm developers and platform operators to adopt the Algorithmic Literacy in the Context of Recommender Systems (ALRS) scale (Sotero et al., 2026) as the basic benchmark for assessing citizens’ algorithmic literacy.

To address cognitive manipulation by recommendation algorithms and its ethical risks under the Destiny Machine metaphor, this paper proposes a governance scheme with clearly assigned responsible actors and feasible strategies (see Figure 1). This scheme makes recommendation mechanisms understandable, substantively empowers users at the technical-tool level to autonomously control the recommendation interface, and combines independent third-party regulation with enhanced citizen digital literacy, building a cognitive immune system effectively guarding against recommendation algorithm manipulation.

**Figure 1:**
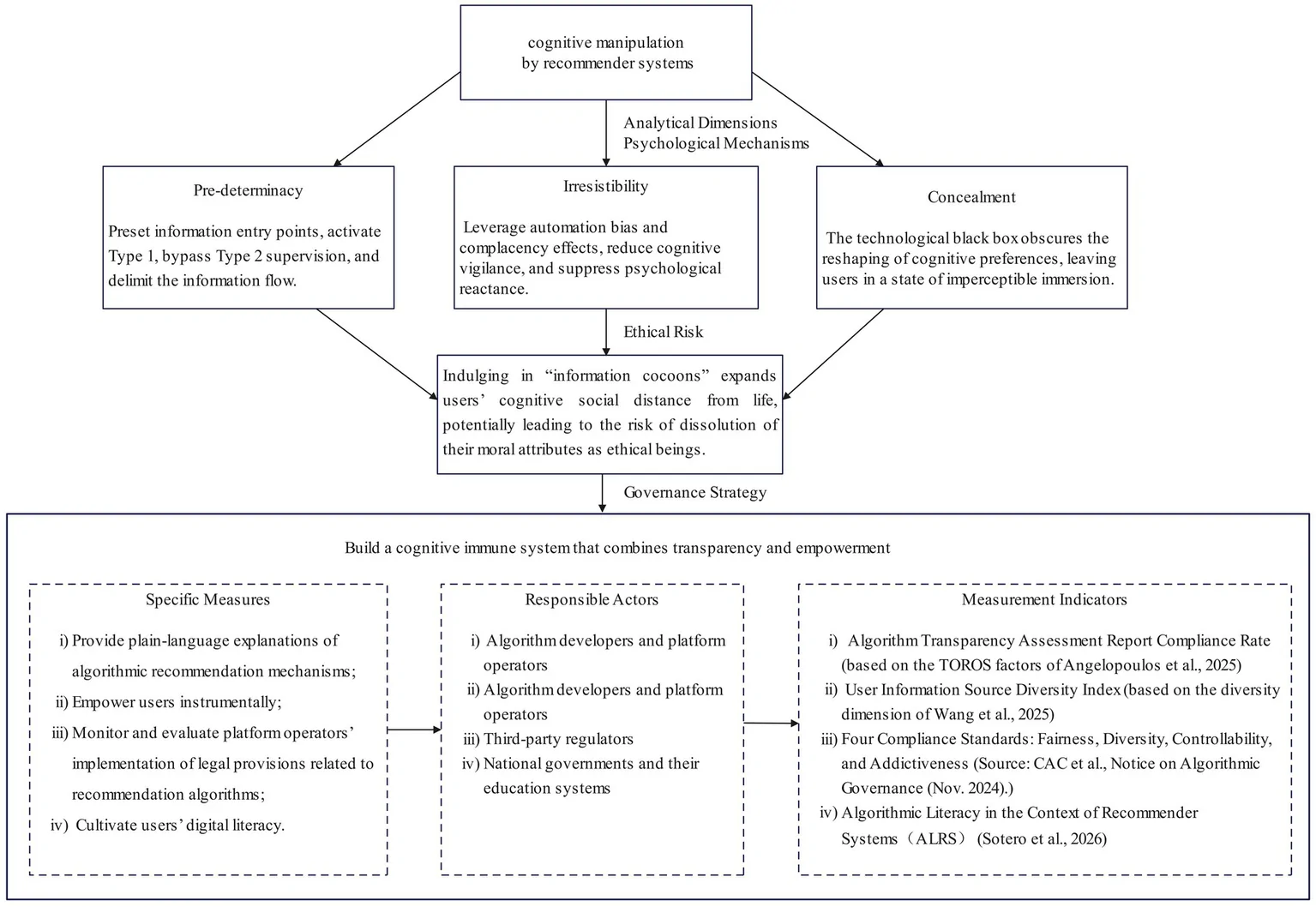
Structural flowchart of cognitive manipulation by recommender systems and its risk governance. Solid-outlined boxes represent the manipulation type, the three analytical dimensions (pre-determinacy, irresistibility, and concealment, each integrating its respective psychological mechanisms), the ethical risks, and the governance strategies. Arrows indicate the progression from the manipulation type to the three analytical dimensions and then to the ethical risks and governance strategies. Within the governance strategy box, three dashed sub-boxes—specific measures, responsible actors, and measurement indicators (in one-to-one correspondence)—are arranged horizontally, with arrows indicating their logical sequence.

## 4 Ethical risks and governance of decision-making manipulation

Autonomous vehicles are gradually moving from technical validation to commercial deployment. According to Fabbri’s (2025) SAP framework, when an autonomous vehicle’s decision system has itself as decision-making subject (S = A) and passengers or pedestrians bear consequences without ability to intervene (A ≠ P), it qualifies as an automated decision-making system (ADMS). By mid-2026, Waymo is the only company globally with a “fully driverless” public narrative and large-scale paid operations across multiple U. S. cities. Its deployed robotaxi fleet exceeds 3,500 vehicles, the largest worldwide, with ridership growing substantially over the past year, completing an average of 500,000 paid trips per week. However, in February 2026, Waymo’s Chief Safety Officer, Mauricio Peña, disclosed for the first time at a U.S. Senate Committee on Commerce, Science, and Transportation hearing that when vehicles encounter unconventional road conditions—construction barriers, traffic officer hand signals, temporary roadblocks, or accident scenes—they must rely on remote assistance personnel for guidance, with a considerable proportion of remote operators based in the Philippines (Waymo Admite, 2026).

This disclosure reveals that decisions made by Waymo’s robotaxis in actual operation fall into three categories by decision-making subject: decisions made solely by the automated decision-making system, decisions made by remote operators with system assistance, and hybrid decisions involving both. Objectively, on the one hand, passengers are unaware of these three decision-making modes and how they are switched, and cannot exit during decision execution; on the other hand, the frequency of remote operator intervention, its specific impact, and its boundary with algorithmic decision-making remain opaque to passengers and regulators alike, which objectively gives rise to the risk of decision-making manipulation.

Against this backdrop, this paper adopts the Destiny Machine metaphor, taking Waymo’s robotaxis as the trolley to examine manipulation in automated decision-making systems’ behavioral decisions in the trolley dilemma. The trolley problem originated in Philippa Foot’s, thought experiment (Foot, 1967). Transplanted into autonomous taxis, its speculative ethical decision-making becomes behavioral decisions based on algorithm-preset rules and remote operators substantively intervening without passengers’ knowledge, constituting decision-making manipulation for passengers. Ethical rules and decision implementation details preset at the design stage are in fact a pre-coding of decisions, potentially risking deconstruction of the individual’s meaning world. To govern this, we argue for a human-machine collaborative decision-making scheme with clear responsible actors, sound measures, and reliable benchmarks under the human-centered approach (see Figure 2).

**Figure 2:**
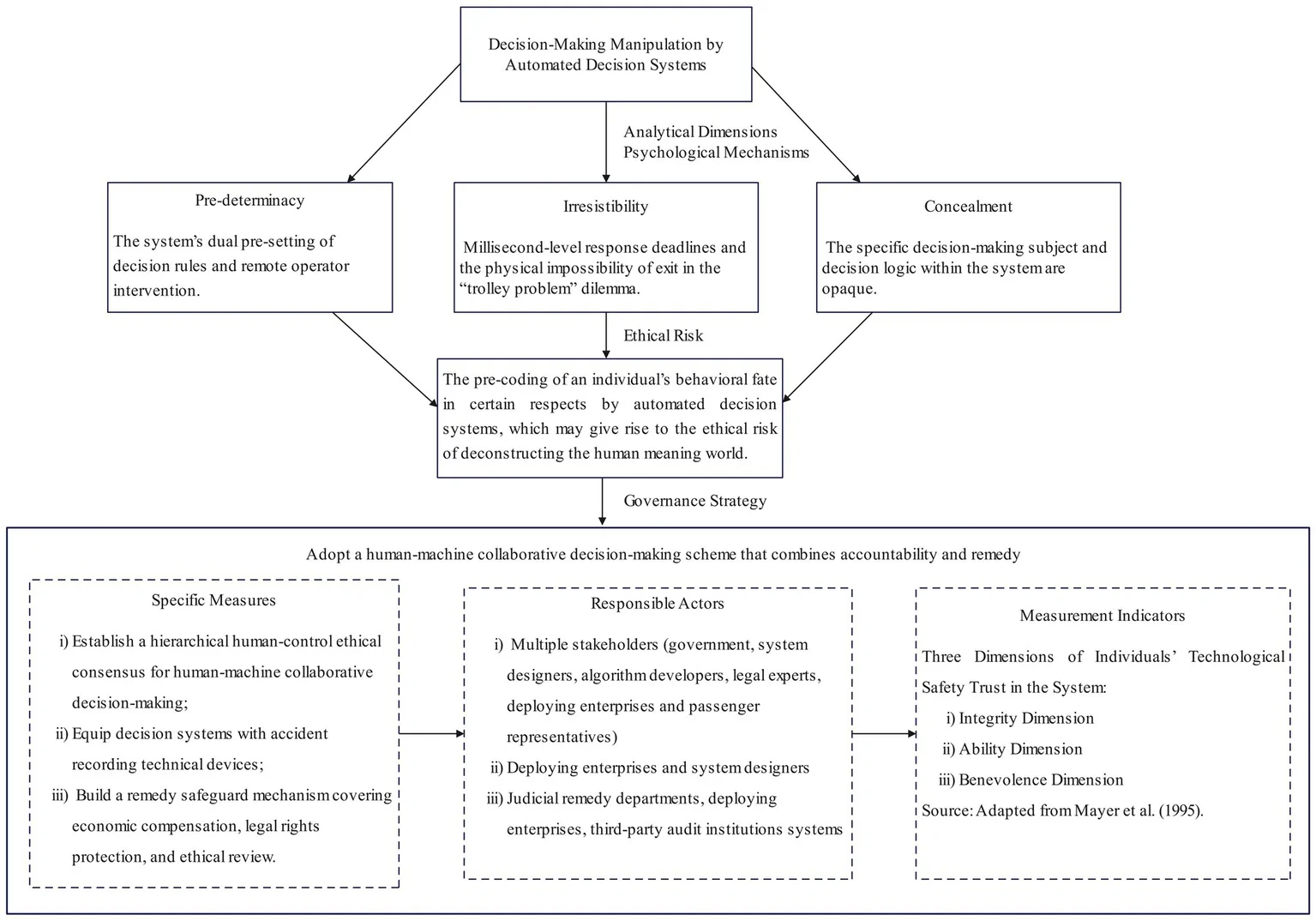
Structural flowchart of decision-making manipulation by automated decision-making systems and its risk governance. Solid-outlined boxes represent the manipulation type, the three analytical dimensions (pre-determinacy, irresistibility, and concealment, each integrating its respective psychological mechanisms), the ethical risks, and the governance strategies. Arrows indicate the progression from the manipulation type to the three analytical dimensions and then to the ethical risks and governance strategies. Within the governance strategy box, three dashed sub-boxes—specific measures, responsible actors, and measurement indicators (in one-to-one correspondence)—are arranged horizontally, with arrows indicating their logical sequence.

### 4.1 Decision-making manipulation by automated decision systems

In the autonomous vehicle trolley problem, a fully autonomous vehicle with brake failure must choose among three exclusive options: hitting a jaywalking pedestrian, hitting a law-abiding passerby, or hitting a wall and killing the passenger. Academic and industry debates identify three strategy types: active (saving the most people, comprehensive scoring, occupant-first protection, outsider-first protection); passive (non-intervention, random selection, braking to a stop); and transfer (ex ante and real-time transfer) (Zheng, 2024). Active strategies more strikingly expose how automated decision systems determine individual fate via pre-set ethical rules. Although the ethical rules pre-set for automated decision systems involve multiple actors—algorithm developers, system designers, ethics experts, legal experts—they ultimately remain a pre-adjudication of the fate of potential parties at the design end, and, at the operational end, remote operator intervention turns the decision-making subject from a single system into a hybrid of system and remote operator. Through pre-determinacy, irresistibility, and concealment and their interrelated psychological mechanisms, this constitutes decision-making manipulation of passengers by automated decision systems.

First, the pre-determinacy of automated decision systems manifests in the dual pre-setting of decision rules and remote operator intervention. On the one hand, active solutions to the trolley problem essentially involve pre-set “harm allocation” rules, i.e., the algorithm predefines crash priorities for robotaxis. Because these rules are determined at the design end and executed during operation, they are difficult to modify or adjust according to real-world scenarios. In other words, decision rules for automated decision systems in the trolley dilemma are pre-programmed at the design end; the lives of passengers involved are simplified into quantifiable weight variables, and their fate has already been set in advance by the system before the accident occurs. On the other hand, the design end allows remote operator intervention, which renders the system’s automated decision-making in the trolley dilemma merely an external technical facade; passengers may experience system-only decisions, system-assisted remote-operator decisions, and hybrid decisions combining both.

For passengers, the system’s dual pre-setting leverages dual-process theory and automation bias at the cognitive level. Passengers tend to rely on Type 1 processing, perceiving the trip as a continuous experience of “the automated decision system driving the vehicle.” Their Type 2 processing, following the law of least effort, lacking cues for rational scrutiny, and with low motivation, typically adopts Type 1 outputs. Passengers are unaware of whether and how system-assisted remote-operator or human-machine hybrid decisions occur; even when aware, they tend to view such modes as safe. This is because automation bias fosters trust in automated decision systems, further strengthening dependence, making errors difficult to correct in time (Romeo and Conti, 2026).

Second, the irresistibility of automated decision systems manifests in millisecond-level response deadlines and physical impossibility of exit in the trolley dilemma. Passengers are virtually powerless to intervene in preset paths—modifying algorithmic ethical rules instantly through moral judgment—with extremely low success likelihood. Because the system directly controls driving, passengers cannot abort the trip; this impossibility stems from enclosed physical space and uninterruptible driving. Waymo’s global head of operations stated in a letter to senators that remote agents only provide suggestions, not directly control, steer, or drive the vehicle. Yet crash consequences borne by passengers are jointly caused by the decision system and remote operators. When decision-making agency is dispersed between system and remote agents, passengers can neither attribute responsibility to either party nor form effective psychological reactance, because the object of resistance is ambiguous.

Finally, concealment in automated decision systems lies in the opacity of the specific decision-making subject and logic, constituting the core dimension of decision-making manipulation. As noted above, the trolley problem involves three decision-making modes. Apart from the system-only mode, the other two embody a contradiction between the “fully driverless” narrative and “remote operator intervention” reality. Passengers know neither the specific decision-making subject nor the algorithm’s decision logic and method of weighting lives. Consequently, passengers tend to view decision outcomes as the system’s unilateral output, with remote operators hidden. This concealment makes potential or ongoing decision-making manipulation appear as a neutral technical operation. Facing collision risk, passengers tend to trust and accept the system’s collision options due to automation bias, Type 1 processing, Type 2 laziness, and cognitive overload from complex traffic conditions.

### 4.2 Pre-coding of decisions and the risk of deconstructing the meaning world

From the Destiny Machine perspective, automated decision systems like robotaxis have algorithmic logic and ethical rules for behavioral decisions pre-set at the design end, essentially a pre-coding of an individual’s behavioral fate in certain respects. Individuals typically exhibit automation bias toward system outputs, engaging Type 1 processing, while complex technical configuration further overloads cognition. This further intensifies the sense of fatalism that automated decision systems impose on individuals’ development in certain respects, potentially giving rise to the ethical risk of deconstructing their meaning world.

As Gao G. X. (2013), p. 69 observes, the future and ideals determine not only our understanding of the present but also of the past; not only the meaning of the present but also the meaning of the past. The essence of the meaning world lies in humanity’s fundamental questioning of life’s meaning. Life’s meaning is grounded in human finitude: immortal gods do not question the meaning of their own existence; existence and death pose no problem for gods, but for humans they constitute an essential problem of life (Gao Z. M., 2013). The meaning world both carries the possibility of transcending finitude and reflects individuals’ deep need to overcome finite limits and pursue infinite transcendence. Ethically, as an ideological form infused with value connotations, the ethical meaning world is intrinsically correlated with ethical life (Yang, 2009). Ethical life is the practical soil from which the meaning world emerges, while the meaning world is the spiritual sublimation of ethical life at the value level.

For robotaxis’ automated decision-making systems, resolving the trolley problem in fact pre-sets passengers’ life-and-death outcome in this extreme scenario, depriving them of autonomous life-and-death choice and the chance to continually question life’s meaning. Furthermore, large-scale commercial use of automated decision-making systems without technical safety assessment and ethical risk review by third-party regulators may potentially extinguish the real flame of individuals’ meaning-world construction. This is because the meaning world is always tied to an individual’s openness toward the future; once any aspect of life is pre-set by such systems, the future of possibilities degenerates into an already-come unidimensionally dominated by technology, and the human meaning world thus faces the ethical risk of deconstruction.

### 4.3 Adopting a human-machine collaborative decision-making scheme combining accountability and remedy

To address decision-making manipulation and its ethical risks, this paper, guided by the human-centered approach, proposes a human-machine collaborative decision-making scheme combining accountability and remedy. Human-machine collaborative decision-making simply means that human decision-makers and intelligent machines cooperate and complement each other’s strengths to jointly complete decision-making tasks and achieve a 1 + 1 > 2 effect (Wang Z. H. et al., 2025). Three main modes exist: first, human decision-makers collaborate with AI by retaining control over decision-making steps (Leyer and Schneider, 2021); second, humans and AI assistants make decisions sequentially, one decision-maker’s output serving as the other’s input—a hybrid sequential decision-making mode (Shrestha et al., 2019); third, AI-assisted human decision-making (Li et al., 2020).

Given the composition of decision-making subjects in robotaxi automated decision systems, direct consequences borne by passengers and pedestrians, and post-accident accountability, this paper advocates the third mode of human-machine collaborative decision-making in the trolley problem. For this mode’s design, we argue that it should draw on the core proposition of the AI-empowered paradigm in the CAFE-AI framework (Suartama et al., 2026): AI is positioned as a tool that can be shaped and controlled by humans, with its role being to assist rather than replace human judgment; humans, at the cognitive level, serve as the “Human in the Loop,” strategically guiding the system’s operation. That is, the system should support rather than erode human autonomy through technical assistance. On this basis, this paper further argues that humans, as the final decision-making subject in the human-machine collaborative decision-making scheme, should bear corresponding legal and ethical responsibility for decision outcomes. Specifically:

First, multiple stakeholders—government, system designers, algorithm developers, legal experts, deploying enterprises, and passenger representatives—should, based on technical validation and actual operations of autonomous vehicles, establish a layered human-control ethical consensus for human-machine collaborative decision-making, ensuring decision-making follows a publicly deliberated ethical model and reserves ample space for passengers’ autonomous judgment. Regarding ethical consensus, Edmond Awad and colleagues found that the global public generally supports saving more lives, saving the young, and saving humans in autonomous vehicle trolley dilemmas, ranked as saving humans > saving more lives > saving the young; they emphasized that although cultural differences are significant across the world, any ethical algorithm ignoring global public moral preferences would struggle to obtain social license (Awad et al., 2018). Such global public moral preferences reflect general or universal basic ethical principles, whose internal value prioritization provides intellectual support for automated decision systems. This experiment also suggests humans should remain cautious toward automated decision systems.

Second, deploying enterprises should work with system designers to equip automated decision-making systems in robotaxis with tamper-proof accident recording devices, drawing on aircraft black box technology. In dilemmas like the trolley problem, neither humans nor automated systems currently have a perfect solution. For automated decision-making systems, since the decision-making subject involves both humans and the system, and direct consequences are borne by humans present, the human-centered approach requires humans to act proactively. Following Lin et al. (2017), we advocate embedding certain safety measures within intelligent machines while allowing humans to serve as supervisory co-decision makers during automatic machine operation. This objectively helps resolve the difficulty of determining responsibility caused by algorithmic black boxes and the non-reproducibility of accidents, providing evidence for ex post accountability.

Finally, judicial remedy departments should work with deploying enterprises and third-party audit institutions to build an ex post remedy safeguard mechanism for human-machine collaborative decision-making, granting accident victims and families economic compensation, legal rights protection, and ethical review. Economic compensation aims to ensure timely compensation for victims and families in both non-compliant and compliant scenarios. Legal rights protection and ethical review should be handled by a third-party audit institution established for human-machine collaborative decision-making. This institution should comprise system design experts, algorithmic technical experts, technology ethics experts, technology law experts, traffic law enforcement practitioners, and public representatives, without conflicts of interest, and be responsible for special investigations of accidents to fairly adjudicate the legal and ethical responsibilities of all parties.

A human-machine collaborative decision-making scheme combining accountability and remedy can provide passengers with “technological safety trust,” which also serves as a benchmark for evaluating the scheme. This trust draws on Mayer et al.’s (1995) integrative model of trust: ability, benevolence, and integrity. For automated decision-making systems in autonomous driving, ability is the system’s precise perception and efficient decision-making in complex traffic scenarios; benevolence is the algorithm’s adherence to life-maximizing ethics in extreme situations; integrity is a decision-making process that is open, transparent, and consistent with public order and good morals. For the human-machine collaborative decision-making scheme, the layered human-control ethical consensus and the publicly deliberated ethical decision-making model correspond to integrity; the tamper-proof recording device corresponds to ability; and the ex post remedy safeguard mechanism comprising economic compensation, legal rights protection, and ethical review corresponds to benevolence.

When accountability mechanisms clarify responsibility and remedy mechanisms guarantee compensation, passengers can overcome blind reliance from automation bias and build rational, prudent, and resilient technological safety trust. This trust can both dissolve the risk of deconstructing the meaning world from preset rules in automated decision-making systems, restoring individuals’ sense of control and dignity over their behavioral fate in certain respects, and provide a psychosocial foundation for such systems to operate on legal and ethical tracks. These three dimensions establish the external conditions and psychological foundation for passengers’ trust in automated decision-making systems. Admittedly, the integrative model of trust was originally developed for organizational trust; this paper transfers it to technological safety trust in autonomous driving, and this transfer’s applicability requires further examination.

## 5 Conclusion and outlook

Under the Destiny Machine metaphor, non-invasive intelligent technology’s manipulation of individuals and its ethical risks are issues humanity must confront. This paper focuses on two currently widely applied non-invasive intelligent technologies, revealing cognitive manipulation by recommendation algorithms and decision-making manipulation by automated decision systems. Comparative analysis shows that recommendation algorithms, via preset information flows and concealed black-box mechanisms, activate users’ Type 1 processing, eroding their reflective capacity in an illusion of empowerment; automated decision systems, via Type 2 cognitive laziness, automation bias, and cognitive overload, obtain outsourced decision power and instantly lock passengers’ life-and-death fate at the behavioral decision level. Together, they form a manipulation path from the external information environment to the behavioral decision level, with a fundamental pre-determinacy logic throughout.

Thus, individuals face dual ethical risks. First, recommendation algorithms’ information cocoons widen social distance between individuals and the lifeworld; users psychologically transfer moral decision responsibility to algorithmic systems, risking dissolution of their moral attributes as ethical beings. Second, an individual’s developmental destiny in certain respects is pre-coded by automated decision systems, putting at risk the meaning world that transcends the finitude of life—a world that may be dismantled by technology. Together, these risks indicate that when technological systems progressively replace individuals’ moral reasoning and autonomous decision-making, individuals’ status as moral subjects is negated by intelligent technological manipulation characterized by pre-determinacy, irresistibility, and concealment.

Facing these challenges, this paper, guided by the human-centered approach, proposes two targeted governance schemes. For cognitive manipulation by recommendation algorithms, we advocate building a cognitive immune system combining transparency and empowerment, establishing and consolidating users’ defensive mode of critical reflection and resistance by enhancing their self-efficacy. For decision-making manipulation by automated decision systems, we advocate a human-machine collaborative decision-making scheme combining accountability and remedy, ensuring technical explainability and controllability, clarifying legal and ethical responsibility attribution and remedy channels, and thereby building individuals’ technological safety trust in automated decision systems.

Notably, with the development of frontier technologies like invasive brain-computer interfaces (BCIs), a more fundamental and serious existential manipulation may emerge. A BCI refers to a direct communication and control pathway established between the brain and external devices centered on computers, using signals generated by the central nervous system, without the mediation of peripheral nerves or muscles (Wolpaw et al., 2000). Existential manipulation based on this technology operates at the level of neuronal activity, bypassing individuals’ autonomous decision-making through pre-conscious detection and reducing them to executors of system instructions. It is characterized by irreversibility after physical embedding in brain tissue, dependence, and concealment. In this regard, this paper argues that “red lines” and a humanistic bottom line for frontier technology applications should be prospectively established to safeguard the integrity of the human species and the development logic of humanistic civilization. It must be acknowledged that such frontier technologies remain at the stage of medical research and clinical trials, have not yet entered commercial application, and relevant empirical evidence is limited. Therefore, this paper treats existential manipulation as a speculative but important future concern, rather than an established consequence of current use of technologies such as invasive BCIs.

The Destiny Machine metaphor warns that intelligent technology’s manipulation of individuals is not an accidental deviation after losing control, but a structural tendency embedded in design logic and operating mechanisms. To this end, a trinity of safeguards—technological, legal, and ethical—should be established for frontier intelligent technologies’ R&D and application, preventing degeneration into a Destiny Machine that arbitrarily manipulates individual fate. Only in this way can humanity achieve a dynamic balance between technological empowerment and humanistic guardianship, ensuring intelligent technology always serves human beings’ all-round development.
